# Evaluation of center of mass estimation for obese using statically equivalent serial chain

**DOI:** 10.1038/s41598-022-26763-1

**Published:** 2022-12-26

**Authors:** Elie Chebel, Burcu Tunc

**Affiliations:** 1 Department of Computer Engineering, Bahcesehir University, Istanbul, 34353 Turkey; 2 Department of Biomedical Engineering, Bahcesehir University, Istanbul, 34353 Turkey

**Keywords:** Biomedical engineering, Rehabilitation

## Abstract

The complex structure of the human body makes its center of mass (CoM) estimation very challenging. The typically used estimation methods usually suffer from large estimation errors when applied to bodies with structural differences. Thus, a reliable estimation method is of utmost importance. In this paper, we present a detailed evaluation of a subject-specific CoM estimation technique named Statically Equivalent Serial Chain (SESC) by investigating its estimation ability over two different groups of subjects (Fit and Obese) in comparison to the segmental analysis method. For this study, we used an IMU-based motion capture system and a force platform to record the joint angles and corresponding center of pressure (CoP) values of twenty-five participants while performing a series of static postures. The root-mean-square errors (RMSE) of SESC’s estimation for both groups showed close and lower mean values, whereas the segmental analysis method showed significantly larger RMSE values in comparison to SESC (*p* < 0.05). In addition, we used the Bland–Altman analysis to evaluate the agreement between the two techniques and the ground truth CoP, which showed the accuracy, precision, and reliability of SESC over both groups. In contrast, the segmental analysis method did not present neither accurate nor precise estimations, as our analysis revealed considerable fixed and proportional biases.

## Introduction

According to the World Health Organization (WHO)^1^, obesity is today one of the most visible but ignored public health problems, which is also increasing at a dangerously alarming rate and is regarded as a worldwide epidemic. This complex condition, defined as excessive or abnormal fat accumulation, puts the lives of hundreds of millions of people at risk, as it has been associated with serious diseases and health conditions such as hypertension, dyslipidemia, coronary heart disease, strokes, osteoarthritis^2^ and many types of cancer^3^. In addition to all these consequences of obesity, this excessive amount of fat reshapes the geometry of the human body thus negatively influencing the biomechanics of daily activities such as sit-to-stand^4^ and walking^5^. For instance, Finkelstein *et al*^6^, state that their findings show a clear association between the probability of sustaining an injury and the body mass index (BMI), and that an increase in the prevalence of obesity will likely increase the occurrences of fall injuries. The occurrence of falling is mostly due to the lack of normal balance control, and it has been addressed in several studies that obesity has demonstrated an adverse effect on balance control among various groups of people ranging from children to the elderly^7–11^. This fact highlights the importance of reliable methods necessary to quantify the balance capabilities of this population, which allows clinicians to perform better assessments and offer more suitable injury prevention strategies.

To quantify the balance capabilities of a human; the path and variation of the center of mass (CoM) is often used, as it is regarded as an indicator of stability and an essential parameter for the human postural control system^12^. Nevertheless, estimating the CoM comes with many challenges and the literature presents various methods to estimate this particular point in space.

One of the most common methods to estimate the CoM is the segmental analysis method, in which the human body is divided into multiple segments, and the positions and orientations of these segments are recorded using a motion capture system. The CoM in this method is then calculated as the weighted sum of the positions of each segment’s CoM which are located according to anthropometric tables most famously the ones published by Winter^13^, Zatsiorsky et al.^14^, and De Leva^15^. This method had proven to be practical in some cases but it is still not reliable in many cases, especially for the ones where it is applied on bodies with internal or external body deformations^16,17^. Another famous estimation technique that has been frequently applied, is the utilization of force sensors and Newtonian equations^18,19^. This technique has been regarded as one of the most reliable CoM estimation methods^20^ and it has been used as the reference/gold standard in comparative studies^16,21^ but still lacked practicality as it requires the use of force platforms, which renders the experimentation area limited to the small surface of the platform. This constraint is a considerable limitation for studies that require a lot of mobility.

Recent techniques have been introduced to enhance the estimation process, most notably those which relied on machine learning^22–26^. In our previous work^27^, we introduced a method to map the joint angles to the CoM position using a deep neural network (DNN). In that study, the CoM used in the training dataset was collected from ‘Fit subjects’ using the segmental analysis method, which can be less accurate in cases where the subjects have different body densities as in the case of obesity. This led us to explore a theoretically more accurate and promising technique called statically equivalent serial chain (SESC), proposed by Cotton et al.^28^. This particular technique, based on the work previously done by Espiau and Boulic^29^ on CoM estimation in robotics, presents a way to estimate the CoM of a branched chain without the need for inertial parameters. Rather than assigning the anthropometric data to each segment of the body, in this approach, an identification phase has to be carried out before the experimentation phase. This identification phase provides a subject-specific vector of parameters analogically equivalent to the anthropometric data needed to estimate the CoM as in the segmentation method. This ‘subject-specificity’ feature theoretically gives SESC the ability to overcome the challenges imposed by atypical body shapes in CoM estimations, which led us to further investigate the SESC CoM technique over the obese population.

SESC CoM estimation technique had been used and validated for different populations over the years, like the fit, elderly and hemiplegic populations, in different studies^30–32^. On the other hand, to our knowledge, its accuracy is not evaluated for the obese population yet. We hypothesize that, theoretically, SESC can overcome the effect of significant body differences, however it still needs to be tested. For this purpose, in this study, we investigated the accuracy of CoM estimations based on the SESC technique over obese subjects, in comparison to its accuracy over fit subjects. Additionally, to show its superiority over the segmental analysis technique we compared our estimation results for both groups, with the estimations based on the segmental analysis technique. To evaluate the estimation performance of those two techniques, in addition to their accuracies, we compared their precision, as well as their agreement with the gold standard reference method.

## Methods

### Participants

Twenty-five adult individuals were elected to participate in the experiments, eleven fit (BMI < 25 kg/m^2^) and fourteen obese participants (BMI > 30 kg/m^2^), with ages ranging between 18 and 30. This study was approved by the Research and Publication Ethics Board of Bahcesehir University and performed at Bahcesehir University Biomedical Engineering Laboratory in Istanbul. All procedures were conformed to the Helsinki Declaration. Informed consent was obtained from all individuals participating in the study.

### Data acquisition

The SESC technique requires the knowledge of joint angles and the corresponding horizontal CoM position during static postures.

For the joint angles, a low-cost and accurate motion capture device MVN Awinda (XSENS Technologies BV, Enschede, The Netherlands), was used. XSENS has been verified in literature for its reliability and concurrent validity and considered suitable for clinical applications^33^. This system, which operates at a 60 Hz sampling frequency, is comprised of 17 inertial measurement units (IMUs), which transform the human body into a 23 segments biomechanical model with 22 joints. The MVN software version 2020.2 was used.

For the horizontal CoM position, we used the BERTEC FP-4060-05-PT force platform, as the CoP recorded by the force platform is regarded as the horizontal projection of the CoM during static conditions.

### Center of mass estimation

In this study, we estimated the human CoM using the SESC method and segmental analysis method (provided by XSENS MVN software) during various static conditions, and compared the estimations with the CoP readings recorded by a force platform.

#### Statically equivalent serial chain

##### Biomechanical design

**Figure 1:**
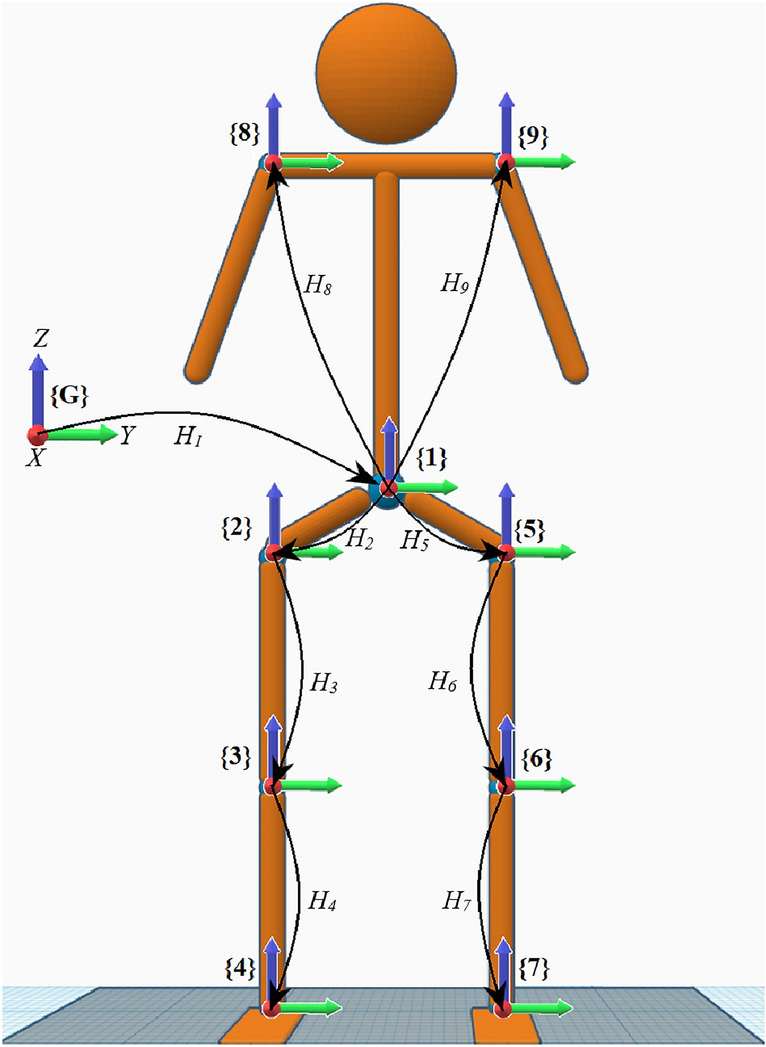
Nine joints—Nineteen DoF biomechanical model. ‘G’ represents the global frame of reference and $H_i$ represents the homogeneous transformation matrix used to describe the spatial relationship between consecutive joints.

The first step in SESC modeling is to design a biomechanical representation of the human body. In this study, a nine-segments nineteen-degrees of freedom (DoF) model is used (Fig. 1). This model is considered to be composed of rigid bodies connected by either spherical (3 DoF) joints representing the shoulders, lumbosacral (L5/S1), and hips, or hinge joints (1 DoF) representing the knees and ankles.

The classical determination of the CoM position of such a structure is computed by calculating the weighted sum of each rigid body’s CoM location as described in Eq. (1).

1 $$\begin{aligned} \overrightarrow{CoM}=\frac{\sum _{i=1}^{n} m_{i} \cdot \vec {C}_{i}}{M} \end{aligned}$$

where $\textit{n}$ is the number of joints, $m_{i}$ is the mass of each segment, $\vec {C}_{i}$ is the location of each segment’s CoM and M is the total mass of the structure $\textit{M} = \sum _{i=1}^{n} m_{i}$.

After expanding Eq. (1), it becomes:

2 $$\begin{aligned} \begin{aligned} \left\{ \begin{array}{c} {\text {COM}}) \\ 1 \end{array}\right\} =&\, \frac{m_1}{M}\left[ \begin{array}{cc} R_1 &{} \vec {d}_1 \\ 0 &{} 1 \end{array}\right] \left\{ \begin{array}{c} \vec {C}_1 \\ 1 \end{array}\right\} +\frac{m_2}{M}\left[ \begin{array}{cc} R_1 &{} \vec {d}_1 \\ 0 &{} 1 \end{array}\right] \left[ \begin{array}{cc} R_2 &{} \vec {d}_2 \\ 0 &{} 1 \end{array}\right] \left\{ \begin{array}{c} \vec {C}_2 \\ 1 \end{array}\right\} \\&+\frac{m_3}{M}\left[ \begin{array}{cc} R_1 &{} \vec {d}_1 \\ 0 &{} 1 \end{array}\right] \left[ \begin{array}{cc} R_2 &{} \vec {d}_2 \\ 0 &{} 1 \end{array}\right] \left[ \begin{array}{cc} R_3 &{} \vec {d}_3 \\ 0 &{} 1 \end{array}\right] \left\{ \begin{array}{c} \vec {C}_3 \\ 1 \end{array}\right\} \\&+\frac{m_4}{M}\left[ \begin{array}{cc} R_1 &{} \vec {d}_1 \\ 0 &{} 1 \end{array}\right] \left[ \begin{array}{cc} R_2 &{} \vec {d}_2 \\ 0 &{} 1 \end{array}\right] \left[ \begin{array}{cc} R_3 &{} \vec {d}_3 \\ 0 &{} 1 \end{array}\right] \left[ \begin{array}{cc} R_4 &{} \vec {d}_4 \\ 0 &{} 1 \end{array}\right] \left\{ \begin{array}{c} \vec {C}_4 \\ 1 \end{array}\right\} \\&+\frac{m_5}{M}\left[ \begin{array}{cc} R_1 &{} \vec {d}_1 \\ 0 &{} 1 \end{array}\right] \left[ \begin{array}{cc} R_5 &{} \vec {d}_5 \\ 0 &{} 1 \end{array}\right] \left\{ \begin{array}{c} \vec {C}_5 \\ 1 \end{array}\right\} \\&+\frac{m_6}{M}\left[ \begin{array}{cc} R_1 &{} \vec {d}_1 \\ 0 &{} 1 \end{array}\right] \left[ \begin{array}{cc} R_5 &{} \vec {d}_5 \\ 0 &{} 1 \end{array}\right] \left[ \begin{array}{cc} R_6 &{} \vec {d}_6 \\ 0 &{} 1 \end{array}\right] \left\{ \begin{array}{c} \vec {C}_6 \\ 1 \end{array}\right\} \\&+\frac{m_7}{M}\left[ \begin{array}{cc} R_1 &{} \vec {d}_1 \\ 0 &{} 1 \end{array}\right] \left[ \begin{array}{cc} R_5 &{} \vec {d}_5 \\ 0 &{} 1 \end{array}\right] \left[ \begin{array}{cc} R_6 &{} \vec {d}_6 \\ 0 &{} 1 \end{array}\right] \left[ \begin{array}{cc} R_7 &{} \vec {d}_7 \\ 0 &{} 1 \end{array}\right] \left\{ \begin{array}{c} \vec {C}_7 \\ 1 \end{array}\right\} \\&+\frac{m_8}{M}\left[ \begin{array}{cc} R_1 &{} \vec {d}_1 \\ 0 &{} 1 \end{array}\right] \left[ \begin{array}{cc} R_8 &{} \vec {d}_8 \\ 0 &{} 1 \end{array}\right] \left\{ \begin{array}{c} \vec {C}_8 \\ 1 \end{array}\right\} +\frac{m_9}{M}\left[ \begin{array}{cc} R_1 &{} \vec {d}_1 \\ 0 &{} 1 \end{array}\right] \left[ \begin{array}{cc} R_9 &{} \vec {d}_9 \\ 0 &{} 1 \end{array}\right] \left\{ \begin{array}{c} \vec {C}_9 \\ 1 \end{array}\right\} \\&\end{aligned} \end{aligned}$$

where, $R_i$ is a 3-by-3 rotation matrix containing the orientation of the segment *i*, and $\vec {d}_i$ represents the 3-by-1 displacement vector between consecutive frames.

##### SESC mathematical modeling

With SESC, the CoM can be located as the end-effector of a virtual serial chain. The architecture of this chain depends on the structure of the biomechanical representation of the whole body. For the branched structure presented in Fig. 1, the serial chain locating its CoM will have the same number of links (9 links) and the same number of DoF (19 DoF). The equation describing this chain (3) can be derived from Eq. (2) after applying the multiplications:

3 $$\begin{aligned} \overrightarrow{CoM}=\vec {d}_{1}+R_{1} \vec {v}_{1}+\cdots +R_{1} R_{9} \vec {v}_{9} \end{aligned}$$

where the vectors $\vec {v}_{1\rightarrow 9}$ contain all of the segments’ constant parameters ($m_i,\vec {C}_i$ and $\vec {d}_i$ ) and *M*.

After reaching the form of Eq. (3), we observe its resemblance to a serial chain comprised of the links $\vec {v}_1$ through $\vec {v}_9$ with the CoM located at its end-effector. This end-effector can be located by applying the following matrix multiplication:

4 $$\begin{aligned} \overrightarrow{CoM}=\left[ \begin{array}{lllll} I&R_{1}&\cdots&R_{1} R_{9} \end{array}\right] \left[ \begin{array}{c} \overrightarrow{d_{1}} \\ \overrightarrow{v_{1}} \\ \vdots \\ \overrightarrow{v_{8}} \\ \overrightarrow{v_{9}} \end{array}\right] =[I {\hat{R}}]\left[ \begin{array}{c} \overrightarrow{d_{1}} \\ \vec {V} \end{array}\right] \end{aligned}$$

where $\textit{I}$ is a 3-by-3 identity matrix, ${\hat{R}}$ represents the 3-by-3n matrix containing the corresponding joint angles, and $\vec {V}$ is the 3n-by-1 subject-specific SESC vector.

##### Identification process

The identification process aims to determine $\vec {V}$ for each subject. Cotton et al proposed that the SESC vector $\vec {V}$ can be calculated using the Moore-Penrose pseudoinverse and partial information of the CoM^28^. In this case, the CoP recorded by the force platform is regarded as the ground projection of the CoM during static conditions (hence the term ‘statically’ in SESC).

Using the pseudoinverse, the SESC vector is calculated as follows:

5 $$\begin{aligned} \vec {V}=[{\hat{\textrm{R}}}]^{\dagger }\left[ \overrightarrow{C O M}-\vec {d}_{1}\right] ={\hat{\textrm{R}}}^{\dagger } \overrightarrow{{\text {CoM}}^{\prime }} \end{aligned}$$

Replacing CoM with the CoP values, and removing the vertical axis-related rows from (5) yields the following form:

6 $$\begin{aligned} \vec {V}=\left[ \begin{array}{l} {\hat{\textrm{R}}}_{{\varvec{x}}} \\ {\hat{\textrm{R}}}_{y} \end{array}\right] ^{\dagger }\left[ \begin{array}{l} {\text {CoP}}_{x}-d_{1, x} \\ {\text {CoP}}_{y}-d_{1, y} \end{array}\right] =({\hat{\textrm{R}}}^{\prime }){^{\dagger }} \overrightarrow{{\text {CoP}}^{\prime }} \end{aligned}$$

For $\hat{R'}$ to be invertible by the pseudoinverse, the number of rows should exceed the number of columns. This requires us to record several distinct joint configurations ‘m’, with ‘m’ being at least equal to 3n/2. Therefore, the equation used to calculate $\vec {V}$ becomes:

7 $$\begin{aligned} \vec {V}=\left[ \begin{array}{c} {\hat{\textrm{R}}}_{x, \textrm{1}} \\ {\hat{\textrm{R}}}_{x, \textrm{1}} \\ \vdots \\ {\hat{\textrm{R}}}_{x, m} \\ {\hat{\textrm{R}}}_{x, {m}} \end{array}\right] ^{\dagger }\left[ \begin{array}{c} {\text {CoP}}_{x, 1}^{\prime } \\ {\text {CoP}}_{y, 1}^{\prime } \\ \vdots \\ {\text {CoP}}_{x, m}^{\prime } \\ {\text {CoP}}_{y, m}^{\prime } \end{array}\right] \end{aligned}$$

##### SESC estimation

After $\vec {V}$ is identified, the full 3-dimensional CoM can be calculated with only joint angles values by using Eq. (4). For more detailed explanations about SESC identification, its calculation, and model simplifications please refer to the following studies^30,34–37^.

#### Segmental analysis CoM estimation

The XSENS MVN system used in this study to obtain joint angles, also estimates CoM positions via the segmental analysis method, by first calculating the orientation and position of each body segment and approximating the mass fractions and CoM positions of each segment taking into consideration the anthropometric tables published in^14,38^. The total body CoM is then calculated using the Eq. (1) previously mentioned in SESC CoM estimation. To compute the segment inertial parameters, such as CoM position and mass fraction, the system uses regression equations and the anthropometric measurements of the subject entered by the user in the “Motion Capture Configuration window”. A total number of twelve body measurements are required in order for MVN to approximate its twenty-three segment biomechanical model. Those measurements are: body height, shoulder height, shoulder width, arm span, wrist span, elbow span, hip height, hip width, knee height, ankle height, foot length, and extra shoe sole thickness (considered zero when barefooted).

For segmental analysis CoM estimations in this study, we used the estimations provided by XSENS MVN system directly, by entering the subject’s body measurements required by the system, and recording the corresponding CoM location for the various static postures.

### Experimental protocol

#### Calibration phase

Before starting the experiments, the participants were asked to put on a specially designed T-shirt that has specific sensor placement landmarks, Velcro straps, and a headband provided by the Awinda system. These wearables were used to hold in place the sensors which were located on the head, shoulders, sternum, upper arms, lower arms, hands, pelvis, upper legs, lower legs, and feet. The sensor placement was carried out as instructed by the XSENS MVN user manual^39^.

A calibration session was followed afterward, which required the subject to stand still for 2.5 s in an upright position called the N-pose, followed by a 3 meters walk from and to the initial standing position. The calibration process was repeated in case it was evaluated as anything less than “successful” by the MVN software. After the motion capture system is calibrated, the participants were asked to stand on the force platform with each foot placed on the marked locations. The marks placed on the force platform represent specific horizontal points known with respect to the force platform’s reference frame. Therefore, by knowing where a subject is standing in both XSENS and the force platform’s coordinate systems, we were able to combine both devices in one single global frame of reference. These two devices were synchronized using a third-party trigger module (Delsys Trigger module). It should be noted that the data collection was performed for each individual in a single take, and was repeated again from the calibration step in case any problems occurrences.

#### Static postures and data preprocessing

The SESC identification phase requires a certain number of joint configurations (postures) along with their respective CoP readings^30^, therefore the subjects were asked to perform a series of distinct postures that focused on the movement of the hips, knees, ankles, the L5/S1, and shoulder joints. The maximum number of postures that we requested from each subject was 120 postures, however, the number of selected postures had an average of 103 due to the difficulties of performing some of the postures as they required a certain level of flexibility. The static postures were selected based on the conditions listed in^40^ , which states that a posture is considered static if, over a window of 1 second, the standard deviation of the angles measurements is less than 1.5° and the standard deviation of the CoP displacement is less than 6 mm. The recorded postures of each subject were divided into two datasets; one was used to calculate the SESC vector (75% of the postures) and the other (25% of the postures) was used to evaluate the estimated CoM. The root-mean-square error (RMSE) was calculated for the evaluation of the estimated CoM.

### Statistical analysis

After calculating the RMSE values for CoM estimations of each subject, the mean, standard deviations and the coefficients of variation of the obtained RMSEs were calculated for each group, and statistical analysis was carried out to investigate the difference in CoM RMSE values between both Fit and Obese groups for both estimation techniques. The analysis was carried out using SPSS (IBM $\circledR$ SPSS $\circledR$ Statistics 25, IBM Corp., USA) software. The conformity of the data to the normal distribution was evaluated using the Shapiro–Wilk test. To compare the Fit and Obese datasets the Student’s t-test was applied in the case of normally distributed data, while the nonparametric Mann-Whitney U test was used for cases that showed a non-normal distribution. For the comparison of SESC and the segmental analysis (XSENS) RMSE results, the paired t-test was used for the case of a normally distributed population while the Wilcoxon signed-rank test was used for the cases that showed a non-normal distribution. The significance level was set as *p* < 0.05. For further assessment of the results, depending on the normality, either Pearson’s correlation coefficient (*r*) or Spearman’s rank correlation coefficient ($r_s$) was calculated to quantify the strength of the linear relationship between the estimated and ground truth (CoP) readings. Furthermore, to investigate whether the estimations based on SESC and the segmental analysis are in agreement with the ground truth readings, we used the Bland–Altman analysis^41^ in which we calculated the mean difference between the estimated and the ground truth values (fixed bias) and 95$\%$ limits of agreemenet (LoA) based on the equation “mean ± 1.96$\times$SD”. In addition, a regression analysis was performed to investigate the existence of a proportional bias^42^. For the regression analysis, because we have multiple estimations from the same subjects, we used longitudinal approach^43^.

## Results

The CoM of each subject was estimated using SESC’s subject-specific vectors, resulting from the identification procedure, and by the segmental analysis method using XSENS’ algorithm. The estimation results are compared to the CoP readings during quiet standing for evaluation.

The resultant RMSE of the CoM estimations by SESC and XSENS’ segmental-based method for each subject of the Fit group, are summarized in Table 1. The RMSE values for the SESC estimations ranged between 9.42 mm and 32.44 mm along the anteroposterior (AP) direction with an average of 18.95 (±6.64) mm, and between 11.34 mm and 21.78 mm along the mediolateral (ML) direction with an average of 17.07 (±3.65) mm. As for the case of the segmental method, the RMSE values ranged between 23.46 mm and 118.80 mm along the AP direction with an average of 51.47 (±23.86) mm, and between 16.07 mm and 71.24 mm along the ML direction with an average of 31.68 (±15.92) mm. It should be noted that SESC also showed a small SD with respect to the mean (CV = 35.03% along the AP axis and CV = 21.37% along the ML axis) in comparison to the segmental method (CV = 46.35% along the AP axis and CV = 50.23% along the ML axis).

**Table 1:** The RMSEs of the CoM estimations based on SESC and segmental (XSENS) method for 11 Fit subjects along AP and ML axes.

Subjects	SESC-Fit		XSENS -Fit	
	RMSE-AP (mm)	RMSE-ML (mm)	RMSE-AP (mm)	RMSE-ML (mm)
1	14.08	21.78	50.62	71.24
2	19.98	15.28	43.31	39.20
3	26.33	19.65	118.80	17.11
4	22.70	14.81	53.06	16.07
5	9.42	11.34	44.91	17.49
6	32.44	18.95	53.01	42.04
7	21.38	15.15	23.46	33.11
8	14.68	11.68	46.18	29.77
9	13.17	17.86	49.86	19.82
10	19.43	19.89	45.23	31.16
11	14.83	21.34	37.71	31.51
Mean	18.95	17.07	51.47	31.68
(SD)	(±6.64)	(±3.65)	(±23.86)	(±15.92)
CV	35.03%	21.37%	46.35%	50.23%

The mean and standard deviation (SD) values of the RMSEs of the subjects and the coefficients of variation (CV) are provided in the last three rows.

The resultant RMSE of the CoM estimations of the Obese group are summarized in Table 2. The RMSE values for the SESC estimations ranged between 8.6 mm and 36.35 mm along the AP direction with an average of 18.20 (±6.97) mm, and between 8.33 mm and 68.12 mm along ML direction with an average of 23.55 (±15.35) mm. For the segmental method (XSENS), the RMSE values ranged between 21.79 mm and 61.03 mm along the AP direction with an average of 38.52 (±12.45) mm, and between 14.03 mm and 52.47 mm along the ML direction with an average of 29.99 (±11.67) mm.

**Table 2:** The RMSEs of the CoM estimations based on SESC and segmental (XSENS) method for 14 Obese subjects along AP and ML axes.

Subjects	SESC-Obese		XSENS -Obese	
	RMSE-AP (mm)	RMSE-ML (mm)	RMSE-AP (mm)	RMSE-ML (mm)
1	8.60	8.33	38.62	40.73
2	12.28	12.52	56.45	19.93
3	20.16	12.08	23.42	14.03
4	14.00	24.32	26.89	31.93
5	21.26	17.22	45.15	29.86
6	17.47	68.12	21.79	51.05
7	13.53	11.60	53.29	20.63
8	36.35	34.77	61.03	52.47
9	13.74	28.98	29.40	32.13
10	18.39	18.78	44.16	20.50
11	22.42	20.58	32.35	33.30
12	13.77	21.42	43.00	23.78
13	16.78	15.27	28.82	19.31
14	26.05	35.75	34.88	30.23
Mean	18.20	23.55	38.52	29.99
(SD)	(±6.97)	(±15.35)	(±12.45)	(±11.67)
CV	38.27%	65.19%	32.31%	38.89%

The mean and standard deviation (SD) values of the RMSEs of the subjects and the coefficients of variation (CV) are provided in the last three rows.

**Figure 2:**
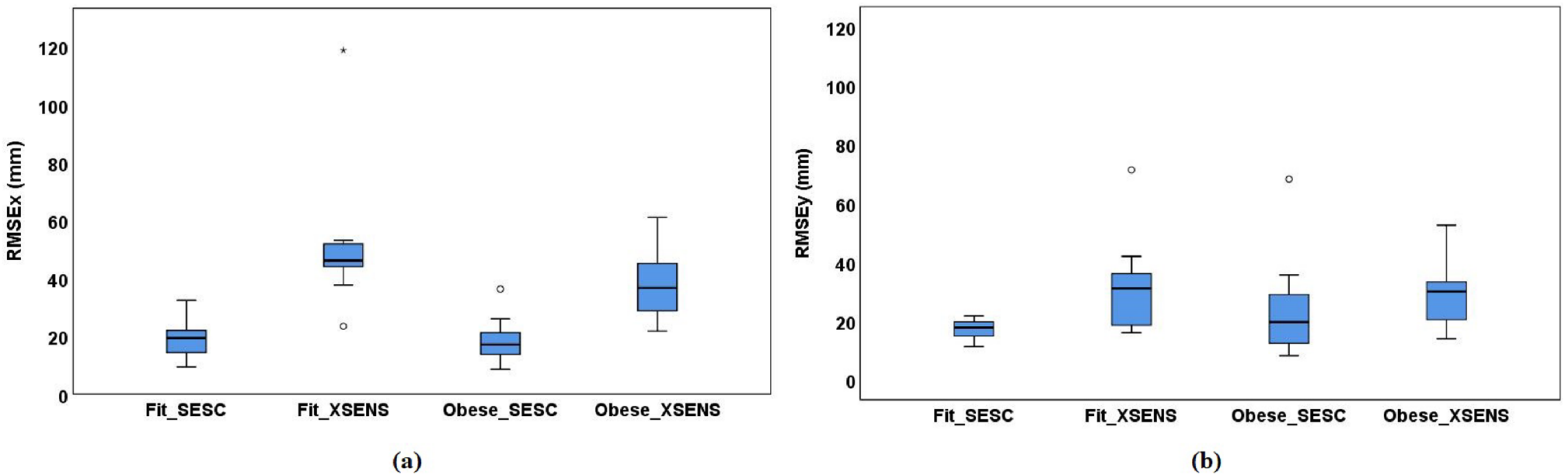
Box and Whisker plots for the RMSE values of SESC and XSENS CoM estimations along the AP-axis (**a**) and ML-axis (**b**) for both Fit and Obese groups. IQRs are represented by the blue boxes, median values are indicated by the bars within the boxes, the whiskers represent the minimum and maximum ranges, and the dots represent the outliers.

The box and whisker plots shown in Fig. 2, present the RMSE values of the CoM estimations based on SESC and XSENS for the Fit and Obese groups. For both groups, along the AP-axis (Fig. 2a), it is clear that the RMSE values belonging to SESC are significantly lower than those of XSENS (*p* = 0.001). On the other hand, there is no statistically significant difference in the comparison of the RMSE values of SESC estimations for the Fit and Obese groups (*p* = 0.788). On the contrary for the XSENS, the RMSE values of the Fit group appear to be significantly higher than those of the Obese group (*p* < 0.05).

The boxplots representing the errors along the ML-axis (Fig. 2b) present different distributions than those of the AP direction. First, by considering the “Fit vs Obese” comparisons, the boxplots show a very close median value (*p* = 0.57 and *p* = 0.83 for SESC and XSENS respectively). However, the data distribution of the Obese subjects (“Obese$\_$SESC”) presents a larger IQR than the one of the Fit subjects (“Fit$\_$SESC”). As for the case of the segmental analysis (XSENS), although the boxplots “Fit$\_$XSENS” and “Obese$\_$XSENS” present a few differences regarding the data distribution and skewness, the overall results indicate a good degree of similarity between them. For the SESC and XSENS comparison, from the plots it is visible that for both Fit and Obese groups, SESC had significantly lower RMSE values than those of XSENS (*p* = 0.005 and *p* = 0.03 for Fit and Obese groups, respectively).

In Table 3, the results of the Pearson correlation coefficients for the CoP measurements and the CoM estimations by SESC and XSENS are given. Those results show strong correlations between the SESC estimations and the ground truth CoP readings for both groups along both axes. On the other hand, for the XSENS CoM estimations, there is strong correlation along the AP axis (*r* = 0.82 and *r* = 0.74 for the Fit and Obese estimations respectively), but weaker correlation along the ML axis (*r* = 0.55 and *r* = 0.49 for the Fit and Obese estimations respectively).

**Table 3:** Pearson correlation coefficients for the CoP measurements and CoM estimations by SESC and XSENS for Fit and Obese groups along the AP and ML axes (Significant correlations (*p* < 0.001) are indicated by *).

	SESC-Fit	XSENS-Fit	SESC-Obese	XSENS-Obese
CoP-AP	0.96*	0.82*	0.93*	0.74*
CoP-ML	0.86*	0.55*	0.75*	0.49*

For further evaluation of the agreement between the estimation methods and ground truth values, we assessed the Bland–Altman analysis. The analysis results are summarized in Table 4.

**Table 4:** Summary of the Bland-Altman variables of each comparison.

Compared methods	Mean difference (Fixed bias) in mm	95 % CI for bias in mm	LoA (upper-lower) in mm	R-squared (*p*-value)	Regression equation
Fit: SESCx vs CoPx	− 0.18	− 1.11 to 0.73	− 34.78 to 34.40	0.012 (0.987)	y = 0.79 − 0.19x
Fit: SESCy vs CoPy	− 1.72	− 2.88 to − 0.55	− 34.10 to 30.66	0.075 (0.039)	y = 1.92 − 0.24x
﻿Fit: XSENSx vs CoPx	− 36.72	− 43.57 to − 29.87	− 104.04 to 30.59	0.430 (< 0.001)	y = − 9.15− 0.43x
﻿Fit: XSENSy vs CoPy	− 16.24	− 24.69 to − 7.78	− 63.75 to 31.26	0.476 (< 0.001)	y = 1.82 − 0.46x
Obese: SESCx vs COPx	− 0.39	− 2.74 to 1.95	− 41.12 to 40.33	0.060 (0.091)	y = 4.55 − 0.06x
﻿Obese: SESCy vs COPy	− 4.60	− 6.71 to − 2.50	− 40.57 to 31.36	0.185 (< 0.001)	y = 2.19 − 0.42x
﻿Obese: XSENSx vs COPx	− 21.15	− 30.81 to − 13.49	− 86.85 to 42.54	0.320 (< 0.001)	y =− 16.09 − 0.27x
﻿Obese: XSENSy vs COPy	− 15.32	− 21.50 to − 9.13	− 63.50 to 32.86	0.384 (< 0.001)	y = − 2.56 − 0.70x

The fixed bias is defined as the mean of the differences between the CoP and the estimated CoM values. The confidence interval (CI) is set at 95$\%$, LoA values are set as mean ± 1.96$\times$SD.

**Figure 3:**
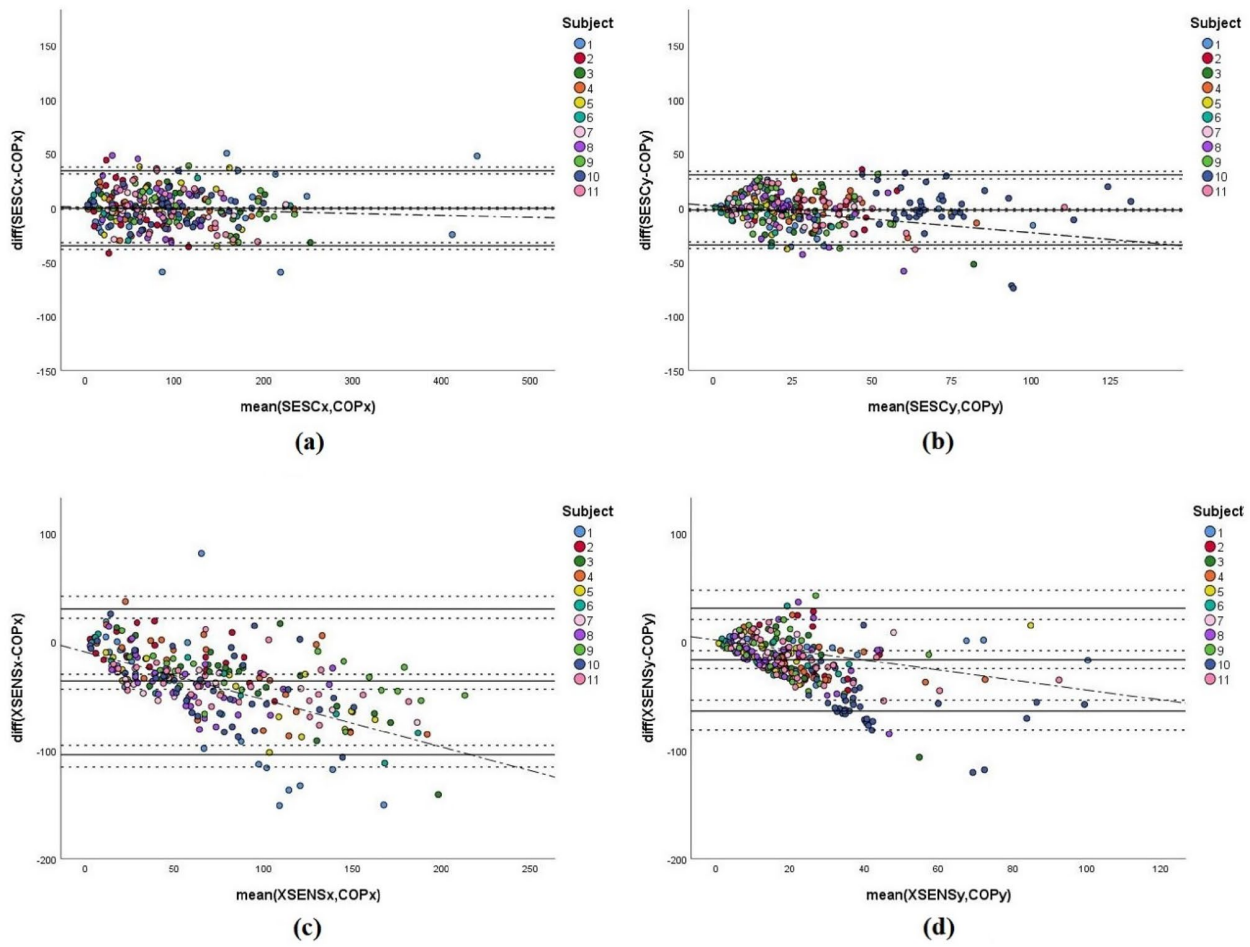
Bland–Altman plots of the CoM estimations against the CoP readings for subjects belonging to the Fit group. Mean bias and limits of agreement are shown by the straight black lines, while confidence intervals are shown by the dotted lines. (**a**) represents the SESC estimations in the AP direction, (**b**) represents the SESC estimations in the ML direction, (**c**) represents the XSENS estimations in the AP direction, and (**d**) represents the SESC estimations in the ML direction.

For the Fit group, the Bland-Altman plots (Fig. 3) show that the data points based on SESC’s estimations (Fig. 3a,b) are scattered close to the zero line with the majority of data points existing within the LoA which ranged from -34.78 mm to 34.4 mm for AP direction (Fig. 3a), and from -34.1 mm to 30.66 mm for the ML direction (Fig. 3b). The mean differences (fixed bias) in the AP and ML directions had values of -0.18 mm and -1.72 mm respectively (Table 4). On the other hand, CoM estimations of XSENS (Fig. 3c,d) present wider ranges of LoA, ranging from -104.04 mm to 30.59 mm (Table 4) in the AP direction (Fig. 3c), and from -63.75 mm to 31.26 mm (Table 4) for the ML direction (Fig. 3d). The fixed bias values are given in Table 4 as -36.72 mm and -16.24 mm in the AP and ML directions respectively.

Furthermore, the scattering of CoM estimations based on XSENS (Fig. 3c,d) shows the existence of a trend described by the 3rd (y = − 9.15 − 0.43x) and 4th (y=1.82-0.46x) equations presented in Table 4.

**Figure 4:**
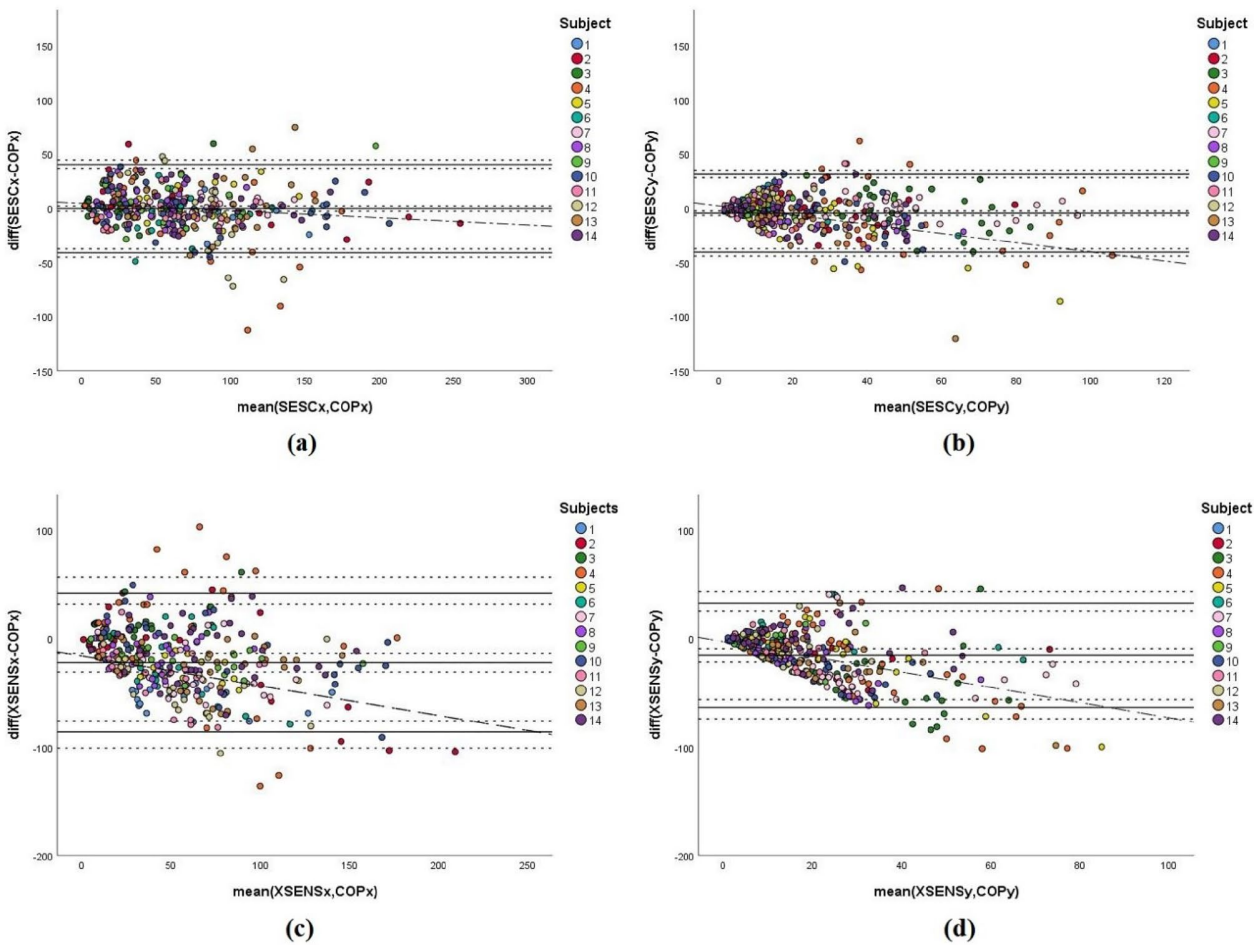
Bland–Altman plots of the CoM estimations against the CoP readings for subjects belonging to the Obese group. Mean bias and limits of agreement are shown by the straight black lines, while confidence intervals are shown by the dotted lines. (**a**) represents the SESC estimations in the AP direction, (**b**) represents the SESC estimations in the ML direction, (**c**) represents the XSENS estimations in the AP direction, and (**d**) represents the SESC estimations in the ML direction.

For the Obese group, similar to the results of the Fit group, the Bland−Altman plots (Fig. 4) show that the data points related to SESC’s estimations (Fig. 4a,b) are also scattered close to the zero line with the majority of data points existing within the LoA which ranged from − 41.12 to 40.33 mm for AP direction, and from − 40.57 to 31.36 mm for the ML direction. The mean differences (fixed biases) for the AP and ML directions had values of − 0.39 mm and − 4.60 mm respectively (Table 4).

On the other hand, XSENS’ data (Fig. 4c,d) present wider ranges of LoA, ranging from − 86.85 to 42.54 mm in the AP direction, and from − 63.50 to 32.86 mm for the ML direction. For the bias values, Table 4 shows that the fixed biases were − 21.15 mm and − 15.32 mm in the AP and ML directions respectively. Furthermore, the scattering of the data points in Fig. 4c and d again show the existence of a trend.

## Discussion

Given the importance of having a reliable CoM estimation technique that can overcome the limitations of the regularly used methods, whether it is being restricted by insufficient space as in the case of using a force platform, or being vulnerable to different body types and weights like the segmental method, SESC is theoretically a strong candidate for CoM estimation. In this study, we conduct an evaluation of the SESC method, in comparison to the segmental analysis method, covering their accuracy, precision, and the ability to maintain a consistent estimation when applied to subjects with significant body differences via evaluating their estimations for Fit and Obese subjects.

Considering the SESC RMSE results, the values of the mean errors shown in Tables 1 and 2 denote a good CoM estimation error for SESC when applied to both Fit and Obese groups. The mean RMSE value along the AP-axis was 18.20 (± 6.97) mm for the Obese group, which is close to the mean RMSE value of the Fit group (18.95 ± 6.64) (*p* = 0.822), thus indicating that along this axis, SESC’s estimation ability was not affected by the change of body structure.

On the other hand, the mean RMSE value along the ML-axis was higher by 6.8 mm for the Obese group. However, this difference is not statistically significant (*p* = 0.57), therefore it shows that the body type change had not affected SESC’s estimation error along this axis as well. Furthermore, it should be noted that the SESC RMSE values along the ML-axis did show a wider dispersion of data for Obese group and a considerably larger SD with respect to the mean (CV = 65.19%), however, in order to properly interpret those results, a larger number of samples should be investigated in addition to a wider diversity of body-weights among the groups.

As for XSENS’ results, along the AP-axis, the RMSE values are considerably high for both Fit and Obese groups (average RMSE values are 51.47 mm and 38.52 mm, respectively). Although the average error for the Obese group was significantly less than for the Fit group (*p* < 0.05), it is, on the other hand, still significantly higher than SESC’s RMSE values (*p* = 0.001). As for the ML-axis, XSENS’ RMSE values showed no statistically significant difference between both groups (*p* = 0.837).

By comparing our SESC RMSE values for both groups to the results presented by González et al.^30^, in which they compared SESC and the Antropometric-based estimations using a gold standard motion capture system (Vicon) and a low-cost device (Kinect), we can determine that our SESC estimations are fairly acceptable for both groups. According to that study, the average SESC-CoM RMSE values ranged from 10.2 to 23.37 mm using both devices, which is comparable to our SESC results, as they fall within the same range.

Regarding the linear correlation between the estimated CoM and the CoP measurements, Table 3 shows that there exists a strong correlation between the SESC estimations and ground truth CoP for both groups in both AP and ML axes. On the other hand, the XSENS estimations show a strong correlation along the AP-axis, and a moderate correlation along the ML-axis. As a result of the strong correlation between the estimation techniques and the ground truth, we conduct a deeper evaluation of those techniques in terms of accuracy and precision.

Using the Bland–Altman method, we further evaluated the agreement between the estimations of each method with the ground truth in both AP and ML axes. According to the plots shown in Fig. 3, we determined that the SESC results for the Fit group (Fig. 3a,b) are in agreement with the ground truth measurements, as the data points are mostly spread within the LoA with no indicator of either fixed or proportional biases. Furthermore, both plots (Fig. 3a,b) show that the mean difference lines are close to ‘0’ indicating a good accuracy in estimation^42^. In addition, these plots also show that the measurements are spread close to each other and the LoA are close to the bias line, thus implying that this technique is precise as well^42^.

On the other hand, Fig. 3c and d, representing the cases for XSENS estimations, show that these values do not agree well with the ground truth readings, as the LoA had larger ranges and CI values than those of SESC’s case (Table 4). The scattering of the data in those plots also indicates a lack of accuracy, as the data points are spread far from the ‘0’ value. Furthermore, we also interpret that there exists a lack of precision with XSENS’ estimations, as the data points scatter away from each other, in contrast to what was seen in the case of SESC. In addition, XSENS’ results clearly present fixed and proportional biases along both AP and ML axes. According to Table 4, the fixed biases (-36.72 mm and -16.24 mm for AP and ML axes, respectively) were significantly large in comparison to SESC results (-0.18 mm and -1.72 mm for AP and ML axes, respectively). Regarding the proportional bias, Table 4 shows that regression models in XSENS’s case have significantly larger $R^2$ values (0.430 and 0.476 for the AP and ML axes, respectively) than SESC (0.012 and 0.075 for the AP and ML axes, respectively), which confirms the existence of a proportional bias in XSENS.

Regarding the Bland-Altman analysis of the CoM estimations for the Obese group, the SESC estimations (Fig. 4a,b) show a good agreement with the ground truth values, similar to the results achieved with the Fit group (Fig. 3a,b), which also confirms the accuracy and precision of SESC. Hence, the general evaluation of the SESC estimations strongly denotes that this method can consistently provide an accurate and precise CoM estimation for humans with different body mass distributions. Nevertheless, we should point out that a slight trend was detected in the plot shown in Fig. 4b indicating a sign of a small proportional bias ($R^2$= 0.185) with SESC’s estimations along the ML axis. This requires further investigations with a larger number of subjects and more variety in BMI especially for the Obese group. As for the segmental method, similar to its results on the Fit group, it still showed an absence of agreement between its estimations and the ground truth values (Fig. 4c,d), in addition to the existence of large fixed biases (− 21.15 mm and − 15.32 mm for AP and ML axes, respectively), and clear indications of proportional biases ($R^2$= 0.320 and $R^2$= 0.384 for AP and ML axes, respectively).

Although the findings of this study give a valuable evaluation of SESC, several limitations should be taken into consideration. First, in this study, we only investigated the CoM estimation along the AP and ML axes without referring to the vertical CoM estimation, as there is still no gold standard method available in the literature, agreed upon by the researchers, that can be used as reference. Second, the evaluation of the CoM in this study is only addressed in static conditions, which is also a considerable limitation that should be further investigated for a better validation of SESC’s estimation capabilities. Additionally, although the number of subjects used in this study was reasonably acceptable, an increase in the number of participants will give a more robust interpretation of the results. Moreover, increasing the biomechanical architecture’s complexity (i.e., increasing the number of segments and DoFs) can further improve the accuracy of the SESC’s CoM estimation. Lastly, our future studies will focus on evaluating the CoM estimation using both SESC and the segmental analysis method over several time periods in order to investigate their test re-test reliability.

## Conclusion

In this paper, we investigated SESC’s CoM estimation ability in overcoming body differences in comparison to the segmental method, by applying it to two groups (Fit and Obese) with different body mass indexes. The SESC’s estimation results were comparable to those mentioned in the literature for both groups and showed complete superiority over the estimation results of the segmental analysis method. In conclusion, we determined that SESC’s accuracy and precision were consistent regardless of the structure of the body under study, and its CoM estimation is suitable for balance assessment for the populations such as the Obese. As for the segmental method, adopted by XSENS software, the results showed significantly larger errors than SESC and a lack of accuracy and precision in CoM estimation for both groups along the AP and ML axes. Further investigations could be carried out to deal with the biases, and offer a better calibration equation to fix the estimation errors.

## Data Availability

The authors declare that the data supporting the findings of this study are available upon reasonable request from the corresponding author and in accordance with applicable regulations and data usage agreements.
